# AUditive Direct in Utero Observation (AUDIO): A Randomized Controlled Trial for a Prenatal Demonstration of Fetal Hearing

**DOI:** 10.3390/diagnostics11112026

**Published:** 2021-11-02

**Authors:** Laura Larcher, Antonio Farina, Danila Morano, Nadia Rimondi, Vincenza Leccese, Elena Contro

**Affiliations:** 1 Division of Obstetrics and Prenatal Medicine, Department of Medicine and Surgery (DIMEC) IRCCS Sant’Orsola-Malpighi, University of Bologna, 40138 Bologna, Italy; antonio.farina@unibo.it (A.F.); nadia.rimondi@aosp.bo.it (N.R.); Vincenza.leccese@aosp.bo.it (V.L.); elena.contro2@unibo.it (E.C.); 2 Department of Obstetrics, Gynecology S. Anna University Hospital, 44124 Cona, Italy; danila.morano@unife.it

**Keywords:** congenital hypoacusis, auditive stimulus, CTG, fetuses, RCT

## Abstract

Introduction: The objective of this randomized controlled study was to demonstrate whether acoustic stimulation in utero is associated with fetal reactivity which is documentable by cardiotocography. Materials and methods: A monocentric randomized controlled trial was performed at a single university tertiary hospital between September 2016 and July 2017. This study was registered as a randomized clinical trial on clinicaltrail.gov (registration number NCT04622059). Unselected pregnancies at term of gestation were consecutively recruited for the purpose of this study. After 10 min of normal cardiotocography without accelerations (non-stress-test with a basal frequency between 110 and 150 beats/min, normal variability between 6 and 15 b/min, no accelerations, and no fetal movements), fetuses were randomized at a 1:1 ratio to either of the two groups. Fetuses in group A (n = 105) received acoustic stimulation after 10 min from the beginning of the CTG, whereas fetuses in group B received no stimulation (n = 105). The outcome variables investigated were the lapse of time between the beginning of the CTG and the occurrence of the first acceleration, and the lapse of time between the beginning of the CTG and the first fetal movement noticed. Results: The lapse of time between the beginning of the CTG and the occurrence of the first acceleration was significantly shorter in the group with acoustic stimulation compared to the other group (14.87 ± 5.01 vs. 21.90 ± 6.94 min, *p*-value < 0.001 log-rank test). Similarly, the lapse of time between the beginning of the CTG and the occurrence of the first fetal movement was significantly shorter in group A compared to group B (17.77 ± 7.62 vs. 23.28 ± 7.61 min, *p*-value < 0.001, log-rank test). Fetal cardiac acceleration and the occurrence of a fetal movement during the first 20 min of the CTG were more frequently recorded in group A compared to group B (respectively, 15% vs. 5% and 20% vs. 8%). Conclusion: This RCT showed an early fetal reaction following auditive stimulus, documentable by cardiotocography. Further research is needed to investigate a possible role of acoustic stimulation in utero for the prenatal diagnosis of congenital hypoacusis.

## 1. Introduction

### 1.1. Background

Congenital hypoacusis has an incidence of 1–3 out of 1000 live births. In comparison to children with standard hearing abilities, infants with hearing loss show an increased risk of impairment in verbal and non-verbal communication, behavioral dysfunctionality reduced psycho-social functioning and decreased educational achievements. Therefore, the early detection of hearing impairment has become extremely important in preventive medicine, since hearing implementation is based on early diagnosis, in order to guarantee a better development of language and higher cognitive functions. Currently, only postnatal screening is available for the diagnosis of hearing impairment.

### 1.2. Aims and Objectives

The aim of the RCT AUditive Direct In utero Observation (AUDIO) was to document the fetal response to an auditory stimulus documentable by CTG as an indirect demonstration of fetal hearing.

## 2. Methods

### 2.1. Study Design

The AUDIO RCT was designed following the CONSORT statement [1]. This AUDIO study was a prospective randomized controlled trial performed between September 2016 and July 2017 at a single university tertiary hospital in Bologna, Italy. Overall, 210 women participated in this study. Unselected pregnancies at term of gestation were consecutively recruited for the purpose of this study.

### 2.2. Patient Involvement

Unselected women at term of pregnancy (37–41 weeks of gestation) undergoing cardiotocography (CTG) according to routine clinical care were consecutively recruited for the purpose of this study. After 10 min of normal cardiotocography without accelerations, (non-stress-test with a basal frequency between 110 and 150 beats/min, normal variability between 6 and 15 b/min, no accelerations, no fetal movements), fetuses were randomly assigned to group A or B following simple randomization procedures (computerized random numbers created by the website www.randomization.com, accessed on 27 July 2017). Fetuses in group A (n = 105) received acoustic stimulation after 10 min from the beginning of the CTG, whereas fetuses in group B received no stimulation (n = 105).

Inclusion criteria were as follows:

- Italian language comprehension;
- Age between 18 and 48 years old;
- Gestational age between 37 and 41 weeks;
- Single fetus in cephalic position.

Exclusion criteria were as follows:

- BMI superior to 30 kg/m^2^ before pregnancy and superior to 35 kg/m^2^ at the end of pregnancy;
- Uterine fibromatosis;
- Premature rupture of membranes or oligohydramnios;
- Fetal anomalies or genetic syndromes;
- Maternal infections in pregnancy (CMV, herpes virus, rubella, syphilis, toxoplasmosis);
- Pathologic CTG or uterine contractions;
- Gestational diabetes with insulin therapy;
- Cholestasis of pregnancy;
- Use of corticosteroids within the last hours;
- Use of prostaglandins for cervical ripening, within the last hours.

### 2.3. Intervention

**Figure 1:**
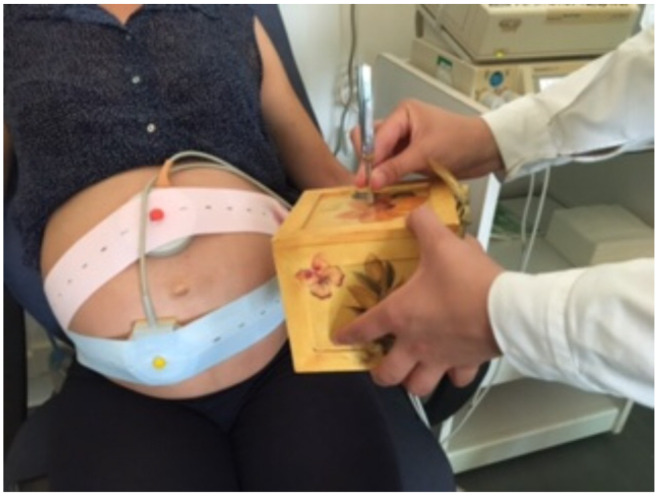
Hearing stimulation procedure in group A: An auditive stimulus was generated in group A. This stimulus was produced by hitting a diapason on an acoustic surface, at a distance of 2–3 cm from the mother’s skin (in order to prevent mechanical vibration reaching the fetus) in the area near the fetus’s head. The same procedure was repeated three times, with 30 s between each stimulus and the following one. The total duration of stimulation was approximately 2 min.

This study was performed in a single quiet room and the CTG was silenced; an auditive stimulus was generated in group A always by the same operator. This stimulus was produced by hitting a diapason on an acoustic surface, at a distance of 2–3 cm from the mother’s skin (in order to prevent mechanical vibration reaching the fetus) in the area near the fetus’s head as shown in Figure 1.

**Figure 2:**
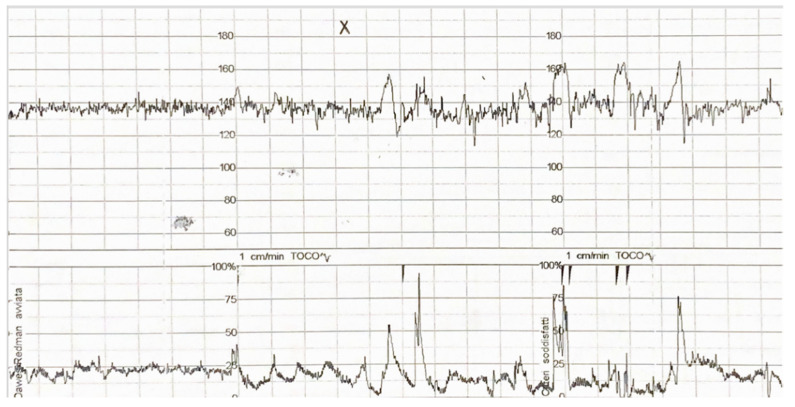
CTG demonstration of fetal reaction: after 10 min of normal cardiotocography without accelerations, fetuses randomized in group A received acoustic stimulation (X). The outcome variables investigated were the lapse of time between the beginning of the CTG and the occurrence of the first acceleration, and the lapse of time between the beginning of the CTG and the first fetal movement noticed (|).

The same procedure was repeated three times, waiting 30 s between one stimulus and the following one. The total duration of stimulation was approximately 2 min. Cardiac accelerations on the CTG and fetal movements perceived by the mother were recorded in the ten minutes after the stimulus was produced. Figure 2 shows the CTG fetal reaction.

No stimulation was produced in group B, however, the CTG was prosecuted until the clinical criteria were satisfied. The total length of the CTG was variable from a minimum of 20 min to a maximum of one hour. 

All clinical variables were recorded in the case report form (CRF), particularly the lapse of time needed for the first fetal cardiac acceleration and for the first fetal movement to occur.

A complete postnatal follow-up was obtained through the examination of medical records and parental interviews. Normal neonatal hearing was documented in all cases according to standard clinical care.

### 2.4. Sample Size

A power analysis was carried out using the Power Analysis Sample Size (PASS) software (Kaysville, UT, USA) and was conducted before the enrollment started. It was estimated that, for a survival comparison test (log Rank), given the sample allocation ratio = 1:1, 100 cases per group would be needed to validate a hazard ratio between group A and group B of 1.5 with a power of 80% and a Type I error of 5%.

### 2.5. Statistical Methods

The Student’s *t* test and χ^2^ test were carried out to make univariate comparisons of quantitative and qualitative variables, respectively, between subgroups.

A Cox regression analysis was used to evaluate the proportion of study participants in the generated subgroups of patients (group A and group B) who experienced the events of interest at a specific time point adjusted for possible confounding factors. The hazard ratio and log-rank test were used to calculate the magnitude of the effects. 

The data were analyzed using the software IBM SPSS Statistical Package for Social Science, Armonk, NY, USA, 2016 (ver. 24.0).

### 2.6. Ethical Approval

The institutional review board approved this study (19 July 2016, number 104/2016/U/Sper), and all participants were provided with and signed a written informed consent form. This study was registered as a randomized clinical trial on clinicaltrail.gov (registration number NCT04622059). This study received no funding.

## 3. Results

A total of 210 women with uncomplicated single-term pregnancies were consecutively enrolled and randomly included in group A or group B. 

A summary of the clinical details and pregnancy outcomes is provided in Table 1.

The characteristics of the CTG variables and fetal movements considered in this study are shown in Table 2. As shown, no significant differences were found for all the demographic variables considered. 

The lapse of time between the beginning of the CTG and the occurrence of the first acceleration was significantly shorter in group A than in group B (14.87 ± 5.01 vs. 21.90 ± 6.94 min, *p*-value < 0.001 log-rank test). Similarly, the lapse of time between the beginning of the CTG and the first fetal movement was significantly shorter in group A than in group B, (17.77 ± 7.62 vs. 23.28 ± 7.61 min, *p*-value < 0.001, log-rank test).

**Figure 3:**
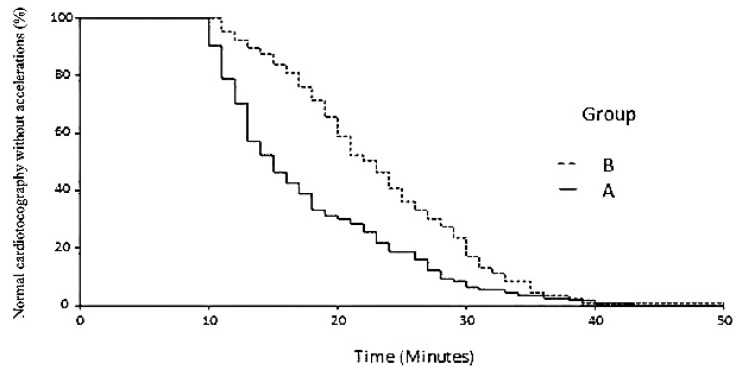
Cox regression plot of elapsed time with normal cardiotocography without accelerations in group A cases (solid line) and in group B controls (dotted line).

**Figure 4:**
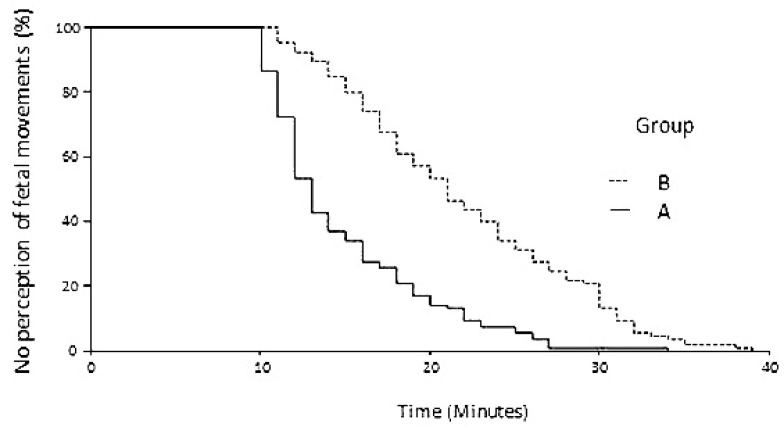
Cox regression plot of elapsed time with no perception of fetal movements in group A cases (solid line) and in group B controls (dotted line).

As shown in Figure 3, the first acceleration occurred in 100% of fetuses in group A after 34 min, whereas 39 min were necessary to observe the first acceleration in 100% of fetuses in group B. Likewise, as shown in Figure 4, for the first fetal movement to occur in 100% of fetuses in group A, 43 min were necessary, vs. 49 min in group B.

The Cox regression analysis did not detect any confounding variables among those considered, and its output is reported in Table 3. As shown, the estimated hazard ratios between group A and group B were 2.81 for the first cardiac acceleration and 1.75 for the first fetal movement; therefore, both were higher than that estimated in the sample size analysis. Routine bootstrap analysis was also reported and confirmed the robustness of the results. Univariable plots showing the probability of observing the event of interest at each time of observation (first cardiac acceleration and the first fetal movement) are given in Figure 3 and Figure 4, respectively. Since we did not observe censor cases, the two curves tended towards 0%, showing that at the end of the period of observation, the events of interest (first cardiac acceleration and the first fetal movement) occurred in all cases of the two series.

## 4. Discussion

### 4.1. Principal Findings

This randomized controlled trial demonstrated an early and reproducible fetal reaction documented by cardiotocography following an auditive stimulus in uncomplicated single pregnancies at term. 

Our RCT was the first study to use a purely auditive stimulus and not a vibroacoustic stimulus in order to obtain a fetal reaction. Some very old studies have been published in the literature reporting a shortening of the CTG testing time and a decrease in false-positive non-reactive CTGs using a vibroacoustic stimulus directly placed on the maternal abdomen [2,3,4,5,6,7,8].

The first one to notice a fetal heart rate acceleration after vibroacoustic stimulation was Bernard in 1947 [9]. In the 1980s, the vibroacoustic stimulation of the fetus with hand-held electronic devices placed directly on the maternal abdomen was widely used [10,11,12]. A prospective randomized clinical trial, very similar to ours, was undertaken in Los Angeles in 1986. In this study, the standard non-stress test was compared with the fetal transabdominal vibroacoustic stimulation with an electronic artificial larynx in order to decrease the incidence of non-reactive CTGs. The authors found a significant decrease (14% vs. 9% *p* 0.004) in non-reactive tests in the study group vs. controls and a significant reduction in testing time [13]. A recent Cochrane review [14] assessed the advantages and disadvantages of the use of fetal vibroacoustic stimulation in addition to other fetal well-being tests and the possibility of decreasing false-positive non-reacting CTGs using this kind of stimulation. The authors concluded that by evoking fetal movements, the vibroacoustic stimulation can reduce the false-positive non-reactive tests due to fetal sleeping and it is therefore useful to optimize perinatal care. Some perplexity regarding the safety of vibroacoustic stimulation was expressed by the authors due to the lack of RCTs in the literature reporting possible fetal consequences (cochlear damage, etc.) of vibroacoustic stimulation. As previously mentioned, in our study, we used a purely acoustic stimulus as the diapason was not directly placed on the maternal skin; therefore, the type of sound produced and the duration of the stimulus (2 min) made the stimulation completely safe for the fetus.

Another important difference between our trial and these previous studies is that we did not aim to reduce the false-positive non-reactive CTGs, as the objective of our study was to document fetal hearing in an objective and reproducible way in order to lay the foundations for an antenatal auditive screening test in the future.

Traditionally, a 20-week fetus is considered capable of perceiving external sounds and react to them, but no clear report of this fetal ability has been objectively documented in the literature. Some studies have tried to demonstrate the development of fetal hearing in utero using ultrasound.

In some studies [15,16] a fetal reaction (fetal movements observed by ultrasound) was obtained in response to a vibroacoustic stimulus. 

However, the main limitation of these studies was the fact that the source of sound was placed to be directly in contact with the maternal skin: Hepper et al. [15] placed a loudspeaker directly on the maternal abdomen; and Das et al. [16] used a fetal acoustic stimulation device over the maternal skin. This way, a mechanical vibration, together with the acoustic wave, reached the fetus. 

### 4.2. Clinical Implication

The clinical impact of the AUDIO study is potentially relevant. Indeed, a prenatal diagnosis of fetal hypoacusis is still not available. An early diagnosis of hearing impairment is very important because it can modify the child’s language development, higher cognitive functions and the social integration. 

### 4.3. Research Implications

If our results are confirmed by further studies, this method, which is easily available at low cost, could be used for prenatal screening in cases with a high risk of congenital hypoacusis (for example, CMV infections or hereditary deafness).

As AUDIO deals with term pregnancies, future research could focus on the second trimester of pregnancy.

### 4.4. Strengths and Limitations

The strengths of our study are as follows: its novelty (only a few and very old studies have been published in the literature and a recent Cochrane review stated the need for further research in this field); the sound methodology (RCT design); and the potential high clinical utility of this easy and economic fetal hearing test. Limitations are possible confounders that should affect the two outcomes of interest; therefore, our results could be overestimated. 

## 5. Conclusions

In conclusion, we may state that the AUDIO clinical trial documented an early fetal response to a purely auditive stimulus which is objective and documentable by CTG. Further research is needed to investigate a possible in utero role of acoustic stimulation for the prenatal diagnosis of congenital hypoacusis.

## Figures and Tables

**Table 1:** Demographic and clinical characteristics of the two cohorts of patients. Data are expressed as the mean (SD) or percentages.

Variable	Group B (n = 105)	Group A (n = 105)	*p*-Value
Maternal age	33.5 (5.55)	33.6 (5.89)	0.942
Gestational age at enrollment	39 (1.15)	39 (1.35)	0.411
% Nulliparae	62.86	64.76	1.00
BMI pre	21.83 (3.39)	21.79 (3.09)	0.810
BMI post	21.83 (3.39)	26.09 (4.05)	0.725
Neonatal weight (grs.)	3321 (461)	3408 (447)	0.167
% of male fetuses	51.43	46.67	0.484
pH	7.29 (0.09)	7.25 (0.10)	0.238
Base excess (BE)	−5.10 (2.25)	−4.89 (3.48)	0.792
% of vaginal delivery	61.90	63.80	0.764

**Table 2:** The characteristics of the CTG variables and fetal movements considered in the study. The total length of the CTG varied according to the clinical criteria.

Variable	Group B (105)	Group A(105)	*p*-Value
Acceleration intervals (min.)	21.9 (6.94)	14.8 (5.01)	<0.001
Number of accelerations	6.40 (3.57)	7.42 (4.60)	0.090
Intervals between fetal movements (min.)	23.28 (7.61)	17.77 (7.62)	<0.001
Number of movements	4.17 (3.22)	5.58 (6.22)	0.082

**Table 3:** Output of Cox regression analysis.

Variable	Hazard Rate *	95% CI		*p*-Value	*p*-Value **
Acceleration intervals (min)	2.81	2.10	3.76	<0.001	0.001
Numbers of accelerations	1.75	1.33	2.30	<0.001	0.001

* Group A vs. group B; ** Bootstrap *p*-value.

## Data Availability

The data presented in this study are available on request from the corresponding author. The data are not publicly available due to privacy.
